# Improving public health data collection approaches across populations: findings from a national evaluation of fruit and vegetable incentives

**DOI:** 10.1017/S1368980025000084

**Published:** 2025-02-26

**Authors:** Carmen Byker Shanks, Betty Izumi, Jenna Eastman, Teala W Alvord, Amy L Yaroch

**Affiliations:** 1 Center for Nutrition & Health Impact, 14301 FNB Parkway, Suite 100, Omaha, NE 68154, USA; 2 OHSU-PSU School of Public Health, Portland, OR 97201, USA; 3 Current institution: Clark County Public Health, Vancouver, WA, USA

**Keywords:** Nutrition incentive, Produce prescription, Dietary screener questionnaire, Dietary assessment

## Abstract

**Objective:**

Public health approaches for addressing diet-related health in the USA include nutrition incentive (NI) and produce prescription (PPR) projects. These projects, funded through the US Department of Agriculture Gus Schumacher Nutrition Incentive Program (GusNIP), aim to support the intake of fruits and vegetables through healthy food incentives. Measuring the GusNIP impact is vital to assessing the ability of incentives to improve public health nutrition outcomes across populations. Shared measures used across GusNIP projects assess fruit and vegetable intake, food security and demographics, among other variables, through a participant survey. This study explored challenges and opportunities to evaluation across populations within a national public health oriented program, GusNIP.

**Design:**

This qualitative study used a sociodemographic survey, semi-structured interviews and focus groups. Descriptive statistics were used to summarise survey data, and applied thematic analysis was used to identify patterns in interview and focus group data.

**Setting:**

Data collection occurred in the USA virtually using Qualtrics and Zoom from fall 2021 to fall 2022.

**Participants:**

Eighteen GusNIP PPR and NI data collectors, twenty-four external evaluators and eleven GusNIP National Training, Technical Assistance, Evaluation, and Information Center staff participated.

**Results:**

Opportunities to improve evaluation among GusNIP’s participants include tailoring surveys to specific subpopulations, translations, culturally appropriate food examples, avoiding stigmatising language, using mixed methods and intentional strategies to enhance representation.

**Conclusion:**

To increase applicability of data collection in public health programs, evaluation tools must reflect the experiences across populations. This study provides insights that can guide future NI, PPR and public health evaluations, helping to more effectively measure and understand outcomes of all communities.

Relatively little progress has been made to meet recommended Dietary Guidelines in the USA, especially in improving diet and related health outcomes^(1)^. The prevalence of diet-related diseases differs by intersecting factors across sociodemographic groups in the USA. For instance, adults who report lower income tend to eat fewer fruits and vegetables than adults reporting higher income, and poverty rates are highest among American Indian/Alaska Native, Black/African American and Hispanic adults and lowest among non-Hispanic White adults. Further, the prevalence of CVD is lower among non-Hispanic White adults compared with non-Hispanic Black adults and Hispanic adults, and similar differences exist for other chronic diseases^(2–15)^.

Public health responses to diet-related health outcomes have centred around programmes that address barriers to affordable, accessible, available and sufficient healthy foods^(10,16,17)^. Programmes like the Special Supplemental Nutrition Program for Women, Infants, and Children (WIC) and the Supplemental Nutrition Assistance Program (SNAP) also play a key role in increasing access to healthy food. WIC provides support to pregnant women, infants and children under five with low income. SNAP, being the largest food assistance programme in the USA, offers broader food assistance to help families supplement their grocery budget.

Further, the 2018 Farm Bill appropriated funds to the Gus Schumacher Nutrition Incentive Program (GusNIP) to support nutrition incentives and produce prescription projects across the USA^(18)^. Through this appropriation, the US Department of Agriculture’s (USDA) National Institute of Food and Agriculture facilitates a competitive grant programme that funds incentive projects to increase access and affordability of fruits and vegetables. GusNIP offers incentives to a wide range of populations, including those who are food insecure, have a low income, have diet-related chronic disease risk and/or face other barriers to healthy food access. Specifically, nutrition incentive projects provide incentives to SNAP and Nutrition Assistance Program participants to purchase fruits and vegetables^(19)^. Produce prescription projects provide incentives or prescriptions to those who are at risk for a diet-related chronic disease and food insecurity (but not necessarily utilising SNAP) for the purchase of reduced cost or free fresh fruits and vegetables^(20)^. Collectively, these types of programmes aim to increase fruit and vegetable intake and promote food and nutrition security, which may serve as a pathway to better health. In addition to funding for nutrition incentive and produce prescription projects, the 2018 Farm Bill allocated funding for the GusNIP Training, Technical Assistance, Evaluation, and Information Center (NTAE) to conduct a national evaluation and support implementation of these projects^(21)^.

Robust measurement tools are necessary to evaluate dietary programmes and interventions, such as GusNIP^(22)^. Case in point, if a programme’s goal is to increase fruit and vegetable intake, then valid and reliable measurement tools should be applied to understand if the programme ultimately achieves this outcome. Ideally, evaluation results can help to tailor an intervention based on a specific population’s needs. This can subsequently improve intervention feasibility, acceptability, appropriateness, implementation, outcomes and impacts.

To ensure evaluation results accurately reflect participant experiences and outcomes, equitable evaluation frameworks emphasise involving all individuals impacted by an intervention at various stages of the process, from preparation and question development to tool adaptation and the dissemination of results^(23–27)^. Approaches to equitable evaluation are wide ranging and adapted for the needs of each project and can include advisory boards, incorporation of the population of interest in developing and reviewing evaluation materials, input on evaluation methods (e.g. qualitative or quantitative), focus on a population of interest, specified terminology applied within a project and assessing communication and dissemination materials for relevance^(23–27)^. In addition, these approaches have elucidated that poorly representative samples and inadequate measurement tools lead to an insufficient and/or inaccurate assessment of the experience and outcomes across populations^(23–27)^. People with low incomes and racial and ethnic populations have been broadly underrepresented in research and evaluation^(9,28)^. Results from socio-economically advantaged and non-minority populations do not necessarily apply to other groups^(29–32)^. Measures developed without intentionally integrating elements from and across populations risk bias and error in outcomes and interpretation of results.

To demonstrate, dietary intake is commonly measured using surveys within nutrition interventions. What people eat is determined by complex interactions of factors at multiple levels: personal (e.g. demographics), social (e.g. peers, family), physical (e.g. neighbourhoods) and macro-level (e.g. policies, culture)^(33)^. These factors ultimately produce distinct dietary outcomes across populations that may (or may not) be captured in research and evaluation results. For instance, because vegetables are often integrated into meat dishes for many cultural or ethnic groups, vegetable consumption may be under or misreported using dietary intake measures that require survey participants to estimate their vegetable consumption separate from meat dishes. Even with the conceptual understanding that representation and adequate evaluation tools are key to understanding the experiences across populations involved in public health nutrition interventions, there is a lack of research that describes practical challenges and opportunities for ensuring equitable evaluation.

## Study goals

This research leverages the NTAE’s work to improve public health data collection approaches in the national GusNIP evaluation of nutrition incentive and produce prescription projects. This study used a qualitative approach with GusNIP data collectors, external evaluators and NTAE staff to explore challenges and opportunities associated with the GusNIP evaluation, which is conducted across many communities and populations.

## Methods

### Background

The co-authors drew upon their experience as researchers and NTAE staff to conduct this study. The NTAE provides reporting, evaluation and technical assistance support to GusNIP grantees, applicants and their partners. The NTAE has guided over 200 grantees to collect shared measures so that an aggregate dataset can be used to understand how GusNIP supports core outcomes. This study focused on the participant-level core outcomes of GusNIP, which include food security, fruit and vegetable intake and perceived health^(34)^. Participant data are collected via surveys and distributed by grantees. The survey assesses fruit and vegetable intake, food security and demographics, among other variables^(35)^. The participant-level survey is typically conducted among a variety of racial and ethnic groups^(30,34)^.

### Study design

The study team applied qualitative techniques with data collectors, external evaluators and NTAE staff from October 2021 to October 2022. This study gathered data through interviews and focus groups, with all participants completing sociodemographic surveys. The research implemented a constructivist approach to understand participant experiences and generate contextual meaning about challenges and opportunities associated with evaluation^(36)^. The University of Nebraska Medical Center Institutional Review Board (IRB) determined that this project does not constitute human subjects research as defined by 45CFR46·102 and did not require further IRB review. Nonetheless, eligible participants provided consent online to participate in the survey and the interview or focus group. Additional verbal consent was obtained prior to recording the interview.

### Recruitment

Data collectors, external evaluators and NTAE staff engaged in focus groups and interviews. To recruit interview and focus group participants, a snowball technique was applied. For data collector interviews, emails were sent to GusNIP grantee contacts inviting study participation and, if interested, to complete a survey. The study staff followed up with eligible interview participants. Next, external evaluators and NTAE staff were recruited via email to an External Evaluators Community of Practice, as well as emails from the NTAE’s directory. An invitation for participation in a focus group was extended, and if interested, the external evaluators and NTAE staff completed a sociodemographic survey and attended one of three scheduled focus groups. For all groups, survey participants were eligible if they were adults (≥ 18 years old) who worked with a GusNIP project as a data collector, external evaluator or NTAE staff. Survey questions also asked about SNAP use and the USDA Food Security module^(37)^. External evaluators and NTAE staff were in separate focus groups.

### Procedures

The semi-structured interview and focus group questions with probes, further described below, were developed by co-authors after reviewing the literature that addresses evaluation elements relevant across populations (Table 1). All interviews and focus groups were conducted via audio-recorded Zoom at a time that was convenient for the participant and researcher. Each interviewee was interviewed one time, and each focus group was conducted one time. The researcher informed the interview or focus group participant(s) about study goals, and consenting occurred before the audio recording began. Co-authors collecting data were trained to conduct interviews and focus groups through their own education in public health research methods, as well as review and practise of the semi-structured guides as a research team. Interviewers discussed data saturation within each group of participants and across the sample as interviews and focus groups were occurring and when they were complete.

**Table 1.** Qualitative question topics asked to participants for interviews and focus groups about evaluation procedures for the Gus Schumacher Nutrition Incentive Program (GusNIP)

*Semi-structured interview topics with data collectors* Project participants in terms of their race and ethnicity Experience working with racial and ethnic populations Challenges when asking participants about: SNAP programme usage, fruit and veg consumption, food security, health, sociodemographics and household characteristics Troubleshooting challenges Translated survey use and challenges Positionality and power dynamics between data collector and survey participants Suggestions for improving the survey or data collection process
*Focus group questions with external evaluators and NTAE staff* Positionality and power dynamics Shortening the distance between the data collector and the participant Cultural appropriateness and relevance of survey items Cultural appropriateness and relevance of survey administration Suggestions for recruiting and training data collectors Improvements to incorporate evaluation across various populations

### Semi-structured interviews with data collectors

A total of eighteen data collectors were eligible and participated. All interviews were conducted by CBS, BI or TWA; only researchers and participants were present during interviews. Data collectors had knowledge of the NTAE and, in turn, potential familiarity with interviewers. Interviews lasted an average of 46 min and ranged from 24 to 120 min. Researchers kept notes while performing interviews. The interview questions focused on experience collecting data across participants, challenges with survey questions, survey language translation, positionality and power and suggestions for improvement. Each participant was offered a $25 gift card incentive for their time.

### Focus group with external evaluators and NTAE staff

Twenty-four external evaluators were eligible and participated in a focus group. The external evaluator focus group was conducted by TWA, with whom the external evaluators were only familiar for the purposes of this research. Eleven NTAE staff were eligible and participated in one of two focus groups. The NTAE staff focus groups were conducted by CBS and TA. NTAE staff had previous relationships with and knowledge of CBS as an NTAE staff member. Across these focus groups, questions concentrated on positionality and power, research protocol, cultural relevance of the survey and data collection process and suggestions for improvement. Only researchers and participants were present during the focus groups. Participants were not offered an incentive since this activity was paid for during time at their job. Focus groups were an average of 60 min in length.

### Analysis

Survey data were entered into Microsoft Excel (version 16) and analysed using descriptive statistics. Interviews and focus groups were transcribed using Rev and then cleaned and deidentified by study staff. The qualitative data were managed using the qualitative software programme Atlas.ti version 7·0 (Scientific Software Development Gmbh). A constructivist approach was used to gain participant perspectives about the challenges and opportunities associated with conducting the GusNIP evaluation across populations^(36)^. Applied thematic analysis guided the coding^(38)^. One researcher coded all transcripts, and two researchers independently split up coding for other transcripts. First, twenty-one transcripts were assigned across the three researchers to independently code. Researchers inductively created a codebook (see Table 2) with codes derived from the independently coded transcripts. The research team applied the codebook to twenty-one transcripts (three focus groups and eighteen interviews) and iteratively updated the codes. Operational definitions and example quotes were added to the final codes in the codebook. Researchers used the final codebook to code all transcripts and focus groups deductively. The coder resolved coding discrepancies by reviewing the transcript with the research team to determine final coding decisions. Codes were collapsed into themes by consensus among researchers. The final codebook included four themes, twenty-two codes and example quotes. The qualitative analysis and results adhere to the COREQ (Consolidated Criteria for Reporting Qualitative Research) guidelines. High-level findings were presented back to the External Evaluators Community of Practice, including participant attendance, to obtain additional feedback. The feedback did not change the codes or themes but supported the interpretation of data within the Discussion section.

**Table 2.** Themes and codes for interviews and focus groups about evaluation procedures for the Gus Schumacher Nutrition Incentive Program (GusNIP)

Theme	Code: operational definition	Illustrative quotes
Survey module challenges	Diet cultural relevance: Dietary Screener Questionnaire (DSQ) questions do not reflect participant’s culture or food ways	I don’t know if it’s because it is like Mexican-type salsa made with tomato that people just like, no, I don’t eat that. And then in terms of the Spanish version, I believe it’s phrased as salsa prepared from home, that’s tomato based. Yeah. And I think there, I think for the Latino community, I think they do eat that sort of salsa. As well as one that’s tomato based, but then there’s also like the green tomato.
	Diet length: DSQ questions and response options were lengthy and caused confusion or frustration	Like common questions that came up like, ‘Oh, what do you mean by that?’ It was, it wasn’t necessarily that confusing. I think like the list of examples of when I was relatively well understood, it was more of like, what’s the, as more kind of like, what’s the point of all of these questions, less. less of an understanding, less about understanding of what is being asked and more about why it’s being asked?
	Diet strategy: Ideas for collecting DSQ questions and responses from participants identifying with racial and ethnic minority groups	So I think it would definitely be nice to see whether like those types of questions being incorporated into surveys or whether new questions can be made up to cater specifically to indigenous communities. Because I just know, like growing up with my parents, you know, our diet sometimes can be a little different from like your traditional Mexican diet, because apart from being Mexican, we’re indigenous.
	Demographic relevance: Suitability of demographic questions across racial and ethnic groups	So the Hispanic community that I personally called the majority of them did pick either prefer not to answer or some other race, and then they were able to fill in, for example, Hispanic or Latino, whatever they preferred. And then some people. They would put their nationality like Mexican instead.
	Food security relevance: Suitability of food security questions across different racial and ethnic groups	I think that those questions don’t always resonate for populations where there’s intergenerational poverty populations who have migrated across various countries. For example, I have talked to a lot of folks who have come to the USA from Mexico and Central America who say, this is great compared to like my experience with food and my country of origin. So, even though I’ve reduced my dietary diversity down to these three foods and I skip meals like things are pretty sweet now.
Factors influencing survey participants’ experience	Stigma: Societal judgement around utilisation of social programmes and/or interventions and associated stigma experienced by participants	There were disagreements on the planning team across the different agencies that implement double up food bucks about whether wearing things that said double up food bucks was a better idea or a worse idea. Some people felt that it was stigmatising and that it would call out people who were doing the survey in a public setting. Other people felt like it was destigmatizing by saying like, hey, we’re all doing this thing.
	Sensitive survey questions: Survey questions may be uncomfortable to answer	I think sometimes people get defensive when that question arises. I believe it might be because of stereotypes that sometimes they feel like, ‘oh, like these people are going to judge me because I receive EBT or SNAP CalFresh and they don’t want to be truthful with their answers.'
	Survey translation and interpretation: Challenges or opportunities related to translated and interpreted surveys	I think the potato ones are maybe a little bit weird in Spanish. They don’t translate as well because like it says like tater tots, and then it is croquetas, from what I recall is like what you give a dog. I mean, it croquetas de papas, right, like a potato, but they might not know what that is; at the same time, it might not really matter.
	Survey fatigue: Hesitation to complete surveys generally, often because the amount of research being conducted in the community is tiring.	A lot of the communities that we’re working with, we’re not the only entities, researchers, anything there, so it can sometimes… We’ve heard from members of the community that sometimes they can feel like they’re like a guinea pig or a lab rat. Just constantly being poked but never really seeing any impact from the work.
	Survey literacy: Survey questions may not be appropriate for low-literacy audience	I’m curious about that, and I’m curious about if there are any ways to improve the way that those questions are phrased. The use of all caps and the this, not this. and the examples and the question and the cumbersome response I think to myself are those like the very best version of a set of questions about fruit and vegetable consumption that are appropriate for this audience.
Intersection of data collector identity and participant identity	Data collector characteristics: Characteristics of the data collector characteristics are important	I’d say, just for me, it’s important to be patient and not judgmental in how you state the questions. It’s important to be sensitive to the population that you’re asking the questions to and being patient.
	Power positionality: Differing levels of power between data collector and participant	I think that because I have to identify as part of (anonymous university), that does get viewed in a certain way. It can be viewed with prestige in a certain community from my understanding is. What I thought would happen is they might feel like we’re looking for them to be having certain issues, like we expect them to have issues, or on the opposite side because we’re asking them of their diet, I also thought that maybe people would feel like they had to say that they’re eating better than they actually are because they might feel guilty. Those are some of the things I had thought that some people could feel based on what we’re asking them as well as the organisation that I’m calling to represent.
	Relationship building: Establishing connections in the community is important for the evaluation	I think it was also really important for (name of data collector) and I to spend a lot of time building relationships with all of the participants, talking to them, getting to know them, sharing stories and reference and resources and ideas, all of those pieces of life to kind of bring it back down.
	Representation in evaluation: Community representation in the evaluation is important	That sometimes like I feel like people relate more to me in that they can, like, bring their guard down. So then they start talking about stuff that I would know what they’re talking about and then I can be able to continue with the questions and be able to gauge what they’re saying so that I can choose the right answer. I think that is very helpful, and I’ve noticed that a lot of my participants is that they can kind of go off with some side conversations that I can relate to.
	Representation challenge: Shared cultural background may lead to assumptions that impact data quality	And I think in that sense being a Latina, who is younger, it definitely gave a different response. In some ways, like there was an assumption being made. There’s an assumption being made where, you know, I would ask one particular question and they’d be like, ‘well, you know’, like, ‘you know what I mean’… And then it was based on me and my interpretation and my position of power within my being the person taking the results. Then leaning that on like my experiences versus what they were experiencing.
	Representation strategy: Strategy for engaging community in evaluation	Well, one strategy, we’re going to try for this coming twenty-two (2022) is working with the programme ambassadors that (organisation name) already has in place. So I don’t know if other organisations that manage these types of grants have a similar position, but they have a handful of people that they either have already or are planning to hire for certain communities as programme ambassadors at ten hours a week to really just be focused on that role of outreach and letting people know about the programme and explaining it and getting store managers and cashiers excited and comfortable and broadened and all of those things.
Strategies to strengthen representation across participants in evaluation	Research challenges: Issues related to methods, including design, instrument, protocol and analysis	The yes or no are easier, I think, than the it’s a frequency five question scale (Likert). I can’t remember the exact. But I think those are slightly more difficult because at times people will respond with, even after giving the prompts on the front end, even if you do that every time, which is also cumbersome, it’s easier to say it once, and then just hope that they fill it in correctly. But even if, even when you was, you know, giving the prompts on the front end of every question, I do think that frequently people would respond with something that wasn’t one of those.
	Survey administration: Challenges or opportunities related to administering mode of survey administration (i.e. self/interviewer, paper/electronic)	And I think that’s the hard part about over the phone versus them seeing it themselves, walking them through it (diet questions) where they can see all their options because most people were thinking mostly per week.
	Survey timing: Timing of when survey is conducted	Do you think you can call me at this exact time? Because that’s when I’m going to be going to the bus to going to my other job, so I’ll have enough time to do this survey.
	Other strategies: Strategies for improving the data collection process	Repeating questions as many times as necessary, and we kind of said that up front when we were starting off the survey was ‘if you need anything repeated, if you need anything explained, we’re happy to’. Particularly around like the food insecurity questions, since those seemed to be so redundant.
	Training: Training needs or strategies	A lot of the times this is the first time that they’ve done it. They’re not really comfortable just talking to people off the street, so in our training we make sure that we tell them, ‘Get to know them a little bit. See how their day is going. Compliment them on something. Just make it more comfortable in general as an environment to collect that data.'
	Volunteers: Using volunteers for data collection may have negative implications	I didn’t use volunteers and I didn’t use interns, I had considered it briefly, but I really wanted a different level of accountability for this activity. And again, for data analysis or for some things where there is not direct interaction with programme participants, I would certainly consider that, or if somebody had a thesis project or something, I think that would be a possibility.

## Results

### Characteristics of participants

To describe participants, sociodemographic surveys were collected from eighteen data collectors who participated in interviews and thirty-five attendees of three focus groups (see Table 3). Most participants identified as women and White.

**Table 3.** Selected demographic survey results for interviewees and focus group participants

Survey question	Response options	Interviewee (*n* 18) responses		Focus group participant (*n* 29)^*^ responses	
		*n*	%	*n*	%
How do you identify your gender?	Woman	16	89 %	25	86 %
	Man	2	11 %	3	10 %
	Non-binary/third gender	0	0 %	1	3 %
Are you of Hispanic, Latino/a, or Spanish origin?	Yes	5	28 %	4	14 %
	No	13	72 %	25	86 %
How would you describe your racial or ethnic background? Check all that apply.	Hispanic and Latino/a/x	5	28 %	3	10 %
	Asian	1	6 %	4	14 %
	Black and African American	3	17 %	3	10 %
	American Indian and Alaska Native	0	0 %	2	7 %
	Middle Eastern and North African	1	6 %	1	3 %
	Native Hawaiian and Pacific Islander	0	0 %	1	3 %
	White or European American	9	50 %	21	72 %
What is the highest level of education you have completed?	Less than a high school diploma	0	0 %	0	0 %
	High school diploma or GED	0	0 %	0	0 %
	Some college, no degree	0	0 %	0	0 %
	Associate degree (e.g. AA, AS)	0	0 %	0	0 %
	Bachelor’s degree (e.g. BA, BS)	8	44 %	5	17 %
	Master’s degree or above	10	56 %	24	83 %

* Thirty-five individuals participated in two focus groups. Six of these also participated in interviews and have been removed from the total (*n* 29) for this column to avoid representing their responses twice. All questions had a response option of ‘Prefer not to answer’ and zero individuals selected this response.

### Qualitative findings by theme

Four themes emerged from interviews and focus groups, including survey module challenges, factors influencing survey participants’ experiences, the intersection of data collector and survey participant identity and strategies to strengthen representation across participants in evaluation.

#### Survey module challenges

The qualitative data about the participant survey centred around survey length, fruit and vegetable intake, food security and demographic modules. Overall, the length of the survey (about thirty questions) was a point of concern, especially for data collectors who administered the survey verbally. One data collector described this concern saying, *‘…There was feedback that it was a really long survey. We did have the stipend, the gift cards, that helps. But still it really ranged…’* (I1).

The relevance of the fruit and vegetable intake questions across populations was a consistent concern among interviewees. The questions, which are derived from the Dietary Screener Questionnaire Fruit and Vegetable module, query about consumption of green leafy vegetables or lettuce salad, fried potatoes, other potatoes, other vegetables, salsa, pizza, tomato sauce, beans, fruit and fruit juice^(39)^. In some cases, the phrasing or naming of foods was unfamiliar to the populations surveyed, and they may have been confused about how to respond. For instance, salads were noted as an area that is interpreted differently across populations. Some survey participants did not consume salad but did eat greens in other forms (e.g. cooked collard greens) and were not sure if they should be identified as a salad or other vegetable.

Data collectors mentioned that many fruits and vegetables frequently eaten among the populations surveyed were not directly asked about, which may have led to underreporting. For example, some Hispanic and Latino/a participants did not know whether canned beans would count as a vegetable or if only raw beans that they soaked and prepared at home would count in a question about beans. Additionally, questions about potatoes were noted to be confusing by several data collectors, as survey participants were not sure whether it was only white potatoes or if they should include sweet potatoes or other types of tubers that were not mentioned in the survey.

Similarly, certain foods, such as salsa and pizza (to assess tomatoes consumed), were asked about but were only relevant to some participants. One data collector said,

‘…I think as a participant, I’d be like, “Why are you asking me about salsa and pizza?” … [we should] have some questions that get swapped in and out, depending on the audience. If we know what this population is more likely to eat’. (I10)

Data collectors sometimes made suggestions about example fruits and vegetables that could be included to increase comprehension of the question and reduce underreporting, such as corn, nopales, pico de gallo, tepary beans and collard greens. One data collector noted,

‘Something else that has come up a lot in our pilot testing is how we frame what is healthy and making sure that we find some more culturally relevant examples of what is healthy, because kale may not be what somebody is the most used to. And there are some great, more culturally specific examples of more emblematic fruits and vegetables, balanced meals, that can feel much more relevant to their history’. (FG1)

Data collectors commonly suggested the ability to alter examples in these questions based on the audience and community being surveyed, which is allowable if a grantee works with the NTAE to ensure fruit and vegetable examples are placed within the correct questions.

Response options about the frequency with which participants consumed foods in the fruit and vegetable module were considered confusing and difficult for participants to recount accurately. Response options for the Dietary Screener Questionnaire module included never, 1 time last month, 2–3 times last month, 1 time per week, 2 times per week, 3–4 times per week, 5–6 times per week, 1 time per d, 2 or more times per d and 2–3 times per d, with the addition of 4–5 times per d and 6 or more times a day for 100 % fruit juice only^(39)^. Some survey participants provided answers in terms of a week, while the options included daily and monthly framing. Other participants were distracted by examples and responded with *‘I eat apples’* or ‘*I hate oranges’* and missed the overall category (fruit) or frequency part of the question. One organisation noted that their team began utilising laminated images of food examples and frequency to help participants think through what was being asked. Another noted rewording or breaking down the number of times per d or week. Overall, the survey’s fruit and vegetable questions and response options were perceived to be too long and, in some cases, repetitive.

A survey participants’ individual experience with food security impacted their understanding of and response to this portion of the survey, which relied on the USDA ERS 6-item Food Security Module^(37)^. Food security questions were highlighted by data collectors as, at times, difficult for participants to understand and sensitive in nature. One data collector recalled,

‘…Some had issues with like we couldn’t afford to eat balanced meals. Well, what do you mean by balanced meals? Like who’s defining that? Like, do you want me to show them My Plate? And, you know, and I don’t know, there’s just lots of room for nuance and interpretation, and lots of ways this could not be giving you meaningful data’. (I10)

In some cases, participant perception of subjective terms on the survey item, such as ‘balanced’ or ‘enough’, made it difficult for participants to respond. One interviewee said,

‘No one ever stopped or didn’t answer, but it was a little harder for them to answer some of the questions because like I set their bar of, “Well, I only eat twice a day, but that’s enough for me”’. (I3)

Data collectors also noted several times that these questions felt repetitive to participants, even leading them to comment things like *‘I feel like you’re not listening’.*

The demographic questions were reported to impact rapport with the data collector during the survey. For example, it was noted by four data collectors that older, Spanish-speaking and/or Hispanic or Latino/a survey participants were uncomfortable being asked their gender when they thought it should be obvious to the data collector. One data collector recalled,

‘As you probably know, people are not as familiar with non-binary and [gender descriptions] in Spanish. They would be like, “I’m obviously a female”, or “I’m obviously a male”. I was like, “I still have to ask the question though”’. (I2)

Others were not able to find a choice with enough specificity for their gender. Both instances led to awkward interactions between the survey participant and data collector. Language was also a consideration within the demographics portion as a Spanish-speaking data collector noted that Spanish does not have a verbatim translation for some of the gender identity terms used frequently in English.

The race and ethnicity self-identification prompts were received differently depending on participant identity. For example, some survey participants did not identify with a separate race or ethnicity. One data collector said,

‘Then when it comes to self-identifying race and ethnicity, it’s really hard for people to distinguish because they were like, “Well, I’m Latino. What else do you want me to tell you?” You have to go through the entire thing of like, “Are you White? Are you Asian, Native, Hawaiian?” All of those. That question’s the one that trips people up’. (I2)

Because the question about Hispanic, Spanish, Latino/a identity was separate from the question about race, some participants chose *‘other’* as their race and filled in Latino/a as they had already answered *‘yes’* to the question before. Alternatively, they responded to the data collector with *‘I’m Latina’* or asked if they were supposed to choose White. Data collectors noted the need for choices for Arab or Middle Eastern survey participants. Another data collector suggested a check all that apply approach that combines the race and ethnicity questions.

#### Factors influencing survey participants’ experience

Interview and focus group attendees reported that the survey participants’ experience during data collection was informed by several factors, including their history with being surveyed, stigma, their language and sensitivity to questions posed.

Survey participant fatigue with being surveyed was discussed by data collectors and focus group attendees. Specifically, survey participants answer the same questions in order to access and maintain benefits, such as SNAP, and additional surveying with similar questions can quickly feel excessive. One focus group attendee elaborated,

‘There’s always all this paperwork one has to fill out. And I think that it’s not relegated to SNAP, but a lot of federal aid programs are inherently extractive. So much information is asked of people, so much vulnerable information is asked of people. And people really need to go through this really robust, extractive, invasive process in order to feed their families and meet their basic requirements’. (FG1)

Strategies mentioned to mitigate survey fatigue included ensuring a personable interaction, returning information to the community being surveyed, communicating *‘What’s in it for them’,* limiting the length of surveys, and not requiring survey completion for programme participation. A focus group attendee also questioned whether the NTAE could access existing demographic records for SNAP survey participants rather than asking this set of questions in the survey.

Data collectors described the stigma experienced by survey participants often related to where the data were collected. Some noted that data collection sites have a negative culture around food assistance programmes that may make participants feel uncomfortable participating in a survey regardless of the identity or behaviour of the data collector. One data collector noted that surveying at brick-and-mortar sites (e.g. grocery stores) was easy for identifying survey participants paying with electronic benefit transfer (EBT) at the register who were eligible to complete the survey. Others felt that data collection at brick-and-mortar sites was stigmatising as the data collector is essentially watching private transactions to see who uses an EBT card. One data collector noted that it was easier to build trust with survey participants at farmers’ markets than other locations because they are already used to the staff and programme branding associated with the GusNIP project. One data collector noted that their organisation discussed wearing branded clothing associated with the GusNIP project, as some thought survey participants may feel stigma and others felt that this would help to normalise the programming. Foods asked about in the survey were also brought up as stigmatising. One data collector noted that, even though the survey does not call any foods ‘unhealthy’, some foods asked about have cultural significance that may lead to feelings of stigma saying, *‘Like the difference between fried potatoes…what you’re getting at?’* (I10).

The participant-level survey is available in ten languages (Arabic, Chinese Simplified, Chinese Traditional, English, French, Korean, Russian, Spanish, Somali, Vietnamese), and survey language considerations for participants were discussed. In general, Spanish translations seemed to be effective, with most data collectors only needing to further explain a handful of questions. For example, the phrase *‘making ends meet’* is an American colloquialism and is not common in Spanish; it was suggested that this phrase would have been better translated as *‘having enough money for the things you need’.* Questions about foods may have been interpreted differently by Spanish speakers due to translation. For example, one collector noted that the Spanish survey says *‘base de tomates’* when referring to salsa. Some survey participants would consider this to only be red tomatoes and not include tomatillos, which are green. Another noted that the Spanish survey asks about *‘croquetas de papas’*, which could be confusing as croquetas are more known as dog food and survey participants might not be familiar with them as potatoes.

In general, it was recommended that no matter the non-English language, survey translation should be completed by a native speaker to improve translation quality. In addition, it would be best if data collectors could speak the language to provide further explanations and answer survey participant questions. It was mentioned that translations can be overly formal or only apply to certain regions or dialects of the language. One collector noted,

‘I just think that’s definitely something that I guess the whole research world definitely needs to take a step back and like, realize that languages vary for every region. Even in English, you know, people have different, for example, slang isn’t particular in the research world, but it’s something that’s different for people, for example, who live in California than those who live in New York’. (I15)

However, it was noted that not having team members who speak languages beyond English was a barrier to including all programme participants.

In addition to language, literacy was discussed as a key component in data collection. Data collectors noted that the phrasing of questions and responses can be confusing, especially for survey participants with a lower literacy level. Some noted that this is a barrier to self-administered surveys, as a data collector is needed to describe what a question is asking. One recommended limiting the words used in questions and responses or even changing the approach to surveys, such as use of emojis or thumbs up or thumbs down in place of words when possible.

Some survey questions were noted as sensitive, challenging for survey participants to answer and impacting the experience. Food security questions sometimes caught participants off guard, caused discomfort or shame, led to over-explanation of circumstances or triggered survey participants to decline to answer. One data collector empathised with those who chose not to answer saying, *‘I don’t know how many people are going to, like, raise their hand and be like “yes, we do without food all the time”’* (I10). Additionally, when asked to describe their own health, some survey participants were noted to express shame or uncertainty, saying things like, *‘I know I should be better’* or *‘It’s whatever you think it is’.* Data collectors additionally noted that sensitivity to any of these questions was increased when data collection was being done in public and recommended techniques such as leaning in, lowering voice, paying attention to survey participants’ body language or allowing survey participants to self-administer certain questions. One suggested adding a *‘preamble’* language to sensitive questions to prepare survey participants and help them to understand the reasons they are being asked.

#### Intersection of data collector identity and survey participant identity

Differences in personal identities between the data collector and the survey participant produced some underlying dissonance during data collection. Generally, if the data collector was seen as an outsider to the community, survey participants have expressed distrust, questioned research goals and asked about why the information was needed. Personal identities such as age, race, ethnicity, gender, spoken language, accent, religion and community shaped the data collection process. Several data collectors noted that age and gender were factors in mitigating any sense of intimidation. For instance, one data collector discussed that younger, female data collectors were seen as the least intimidating.

Residential neighbourhood and data collectors’ profession (e.g. employee at a university or local organisation) were also important factors to consider during the data collection process. A data collector said that one could *‘never fully understand’* the impact of a White individual collecting surveys in primarily Black neighbourhoods. The same data collector reported that showing up in a neighbourhood with a clipboard, collecting personal information and then leaving can lead to negative feelings about the research process. Another commented on this saying,

‘If you’re not a member of the community, whether because you’re known by name or you share similar social identity, there’s a hesitancy to engage in conversation with folks, and it kind of played out with this one specifically is, there are some folks that are very, very interested and open to doing the survey, but the majority I’ve found where, one of their original first questions was, you know, “What’s this for, what’s it being used for?”’. (I12)

Similarly, data collector professional affiliation was mentioned as influential on the interaction with the survey participant. For example, when data collectors were required to wear shirts to represent the university for which they were collecting data, survey participants did not have to ask their usual first questions, *‘Who are you and where are you from?’* However, representation from certain institutions (often universities) could lead to survey participants feeling intimidated and/or giving the types of responses they believed were desired.

Similarities between the survey participant and data collector were identified as an opportunity and challenge in evaluation. Data collectors noted that survey participants were more willing to participate if they recognised the person surveying as reflecting their own identity, including race, socio-economic status, language and other characteristics such as age or gender. Having community members who were also nutrition incentive or produce prescription participants on the evaluation team proved pivotal in certain cases. According to this study’s survey data, most data collectors were not food insecure, which may have further distanced the experience with the survey participant.

Sharing a language between data collectors and survey participants increased survey participants’ overall comfort and sense of camaraderie with data collectors. Several data collectors noted that they believed the ability to speak in the language of survey participants increased willingness to participate in the survey process. For example, knowledge of a survey participant’s language sometimes overlapped with knowledge about cultural food ways, providing the data collector with a sense of which food examples on the survey would be relevant. Shared language allowed the survey participant to provide the full and intended meaning in their responses, clarify responses and ask or answer questions about the survey. This was not possible when a translated survey was provided and/or a data collector who could speak the language was unavailable. One data collector who conducted phone surveys noted that although they did not specify their identity, several survey participants seemed to pick up their Latino identity from their voice when speaking Spanish or their full name, which was stated. One data collector noted that sharing a language could be a challenge as survey participants leaned on the data collector too heavily and provided broad responses, such as *‘Well, you know what I mean’.* This specifically occurred with prompts about legumes and salsa where survey participants indicated that they consumed *‘a lot’* of salsa, and the data collector would need to prompt for more specific responses such as, *‘You said you have it with every meal, how many times a day is that for you?’* (I9).

Regardless of intersecting identities between the data collector and survey participant, it was important for the data collector to engage with survey participants in a friendly and conversational rather than formal way. Examples included building rapport by complimenting the participant with comments like ‘*You’re not 75!’* or downplaying the formality of the situation with ‘*I’m just trying to ask you some questions and give you a gift card, that’s all I’m trying to do’* (I11). In addition, the questions needed to be asked in a non-judgmental way to increase participant comfort. Reinforcing that the survey was voluntary and the participant could stop at any time put survey participants at ease. One data collector also noted that truly caring about the project and its participants can really come across to those being surveyed and increase their interest in participation.

Power and positionality between the data collector and survey participants was a commonly discussed theme in both interviews and focus groups. Data collectors discussed that their institutional affiliation, whether a university or simply as a part of the GusNIP project, could lead to survey participants feeling obligated to complete surveys, as well as a sense that there was a right answer that the data collector wanted for each question. One external evaluator said, *‘I just think we are recognizing that the power dynamic exists and we’re asking for feedback on a program and that feedback might tend to be more favorable just because we’re offering incentive for participation’* (FG1). A data collector noted that survey participants, more commonly those identifying as women, made comments such as, *‘I guess I should eat more vegetables’* or *‘That probably has too much sugar’.* Data collectors also discussed the benefits of having specific evaluation staff *v*. utilising staff that survey participants may already know like farmers market managers. The latter may increase rapport and relationship building, but separate evaluation staff may increase comfort via a sense of anonymity with more sensitive questions.

Data collectors noted the benefits of building relationships with community organisations that have existing rapport with intended audiences/communities for data collection. One noted, however, that this strategy proved difficult as most of the organisations were already spread too thin and unable to take on this evaluation, or its outreach efforts, as an additional project. An NTAE focus group member noted the importance of taking on the onus of reaching the communities we intend to represent. The onus should not be on our intended audiences, and they should not be considered *‘difficult to reach’.*

#### Strategies to strengthen representation across participants in evaluation

Data collectors discussed challenges and strategies to *‘shorten the gap’* between data collectors and survey participants, with the goal of strengthening the representation of participants in the evaluation. In some cases, these strategies had been implemented or planned, and others were presented as ideas that may improve data collection with survey participants.

Challenges to research and data collection are centred around financial concerns. Enhancing funding for evaluation specifically would support GusNIP grantee capacity to perform the level of research they desired, and especially to approach data collection across populations. Collecting surveys was perceived by some as an administrative burden, and that was not of enough benefit to the grantee in comparison to the work required. Focus group members agreed that what is considered evidence-based or validated can limit the ability to expand or clarify questions and responses. One elaborated, *‘And I think the problem becomes when we keep using a metric just because it’s been validated, without looking at who it’s been validated for’* (FG1). Others discussed how the limitations of survey questions, such as cultural relevance, are a problem derived from larger public health constructs, like the Dietary Guidelines for Americans or how food security is conceptualised. Others noted limitations to what survey results can say when questions were asked about related but not primary constructs such as barriers to fruit and vegetable intake. Additionally, though, reducing survey length was mentioned as a priority by other focus group members when additional questions were suggested.

Most data collectors completed data collection in person within several contexts, such as farmers’ markets, at brick-and-mortar sites (e.g. grocery and corner stores) and in-home. Data collectors noted the benefits of in-person data collection to include *‘putting a face to the voice’,* building rapport, assisting with issues or questions (even when self-administered), speed of completion, ability of participants to see the questions (as opposed to over the phone) and greater agreement to complete the survey. Those who completed the survey via the phone noted several challenges, including more difficulty in clarifying fruit and vegetable measurements (could not show images for cups) and some difficulty reaching participants who originally had agreed to participate. One data collector noted that difficulty reaching participants was more common with communities with varied identities, which could skew their sample. Another noted that participants who responded on the phone skewed older and that phone surveys could be challenging for those who are hard of hearing. However, it was also noted that phone data collection could improve privacy for participants and reduce pressure to participate. Several also completed data collection via the internet by collecting participant emails or by sharing links and QR codes. Survey invitations or stipends going to spam was a common problem.

Survey timing was a significant factor in survey participation among all potential participants. Some individuals did not have time when asked to participate in the survey and may have been more likely to respond given notice of the survey. Data collectors mentioned that when participants asked about or saw the length of the survey, it deterred them from participation. In store settings, it was helpful to catch survey participants on their way into the store rather than when they were trying to leave. One data collector noted success during busy times, being able to collect 100 survey responses in six visits outside corner stores during morning and lunch rush. Another mentioned the importance of meeting individuals *‘where they are’* rather than adding another errand to complete the survey. It was mentioned that collecting surveys during working hours may skew data toward non-working participants.

Training for data collectors was discussed to improve the survey experience. Organisations often provided training for their data collectors, especially if they were not researchers and were volunteers or students. Interviewees noted the importance of teaching the basics of consent and impartiality to inexperienced data collectors. One organisation brought in university students to help with data collection and provided training for them, including on Zoom and shadowing onsite. Another trained volunteer with a 2-h *‘crash course’* on best practices conducting surveys. Data collectors wanted more information from the NTAE about adding content or clarification to the survey when administering (e.g. add to the list of fruit and vegetable examples or to provide alternate wording to confusing questions). They expressed that this would empower the data collector to *‘read the room’* in the way they present questions.

Several participants discussed the importance of appropriate compensation at all levels of the survey process. This included compensation for survey participants reflecting the time they are spending and information they provide (the wisdom of their experience as a programme participant). There was a preference for physical gift cards (*v*. e-gift cards) among less technically savvy survey participants. One focus group attendee noted that it does not make sense to expect someone to spend time doing something for free, especially if that person is experiencing low income. They elaborated, saying, *‘…until we actually put money behind what we’re saying, which money, it represents power, it represents where we put value in our culture…’* (FG2). Compensation considerations were also important for data collectors, including students and community health workers.

Offering introductory language for the survey was another approach discussed. One focus group attendee recommended starting with an *‘icebreaker’* to simply ask how the programme is for the participant, which would *‘warm up’* the interaction and provide valuable qualitative feedback. Several interviewees and focus group attendees suggested framing the survey goal as a programme improvement tool, not a judgement about the survey participant. It was further recommended to introduce sensitive questions and any set of questions to avoid catching survey participants off guard and reduce uncomfortable reactions. Letting survey participants know that repeating questions as many times as they needed was fine put them at ease when trying to comprehend questions. Finally, survey participants should be clear that they could end the survey at any time.

Representing the GusNIP nutrition incentive or produce prescription programme was a key recommendation when collecting data from survey participants. Some approached this by partnering with community ambassadors to provide verbal information, and others used branded signage and clothing. Printed visuals of EBT cards and nutrition incentive or produce prescription project cards were useful. Using the project name that survey participants would most recognise such as ‘veggie programme’ was important, even if it was different than the official name.

Considering how fruit and vegetable questions and examples may perpetuate ideas around what GusNIP can be used for (fruits and vegetables) was recommended. This concept was expanded upon with one focus group member suggesting the NTAE look beyond the dominant cultural idea of *‘healthy’* food and consider foods that allow people to be connected or reconnected to food that is important to their culture. Further, it was important that examples were connected to the ways that incentives can promote access to these foods.

Strategies to assist grantees in the data collection process were also discussed. Tailoring required sample sizes to meet research needs while considering the burden on smaller, low-capacity sites was suggested. The current required sample size is around 100 cross-sectional surveys annually for nutrition incentive projects and around 100 matched pre and follow-up surveys over the grant award for produce prescription projects. Additionally, more explanation about the importance of validated survey questions would be helpful. It was important to study participants that grantees can share the survey results with the communities where they completed data collection. It was noted that the NTAE currently analyses grantee data and shares it back for them to decide how to disseminate it in their own communities.

## Discussion

**Figure. 1:**
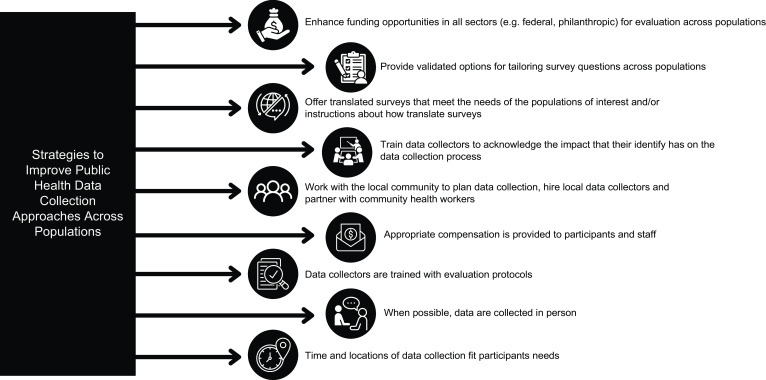
Improving public health data collection approaches across populations: findings from a national evaluation of fruit and vegetable incentives.

Building upon equitable evaluation work being led by the NTAE, we identified challenges and opportunities associated with the GusNIP evaluation across populations in nutrition incentive and produce prescription programmes (see Fig. 1). Emergent themes underscored that data collection was shaped by survey module items, the survey participants’ prior experiences, differences and similarities between the data collector and survey participant and the application of intentional strategies to enhance representation of populations in evaluation. Challenges varied widely from the survey length, culturally unfamiliar food examples, complicated response options, stigma and sensitivity surrounding the survey topics, as well as differing identities between the data collector and participant. A tension existed that underlined these challenges – the need to use validated tools in public health nutrition evaluation and the misalignment of the validated tools with the communities being surveyed. The overall question was raised in multiple ways throughout the study: if a validated tool yields issues with comprehension within the community where it is being used, who is it validated for and who is it not? Equitable evaluation frameworks promote that to accurately understand intervention and health outcomes with survey data, it is key to ensure that further validation of survey tools is conducted to address challenges that emerge during data collection and tailor measures so that they are appropriate across populations^(23–27)^.

While the NTAE’s comprehensive evaluation was acknowledged as essential to understand public health impact, the participant survey compelled programmes to dedicate their available budget to completing the sample size requirements with limited funds. Enhancing funding to grantees for evaluation specifically would provide additional capacity to focus efforts on equitable evaluation. For instance, some GusNIP grantees are community-based organisations that have limited research and evaluation experience or capacity. Reduced capacity for evaluation also led to relying on data collectors from outside of the participant’s community, which influenced the participant’s survey experience. Across public health nutrition practice, increasing capacity to deliver and evaluate programmes requires that many factors are considered: human, financial and infrastructure resources; knowledge to develop strategies and resolve issues; leadership; partnerships; project management; engagement with communities; and workforce capacity and competency to deliver the programme^(40)^.

Several strategies were indicated to better centre populations during survey development and administration. Specific issues with the Dietary Screener Questionnaire Fruit and Vegetable module^(35,36)^, USDA ERS Household Food Security Survey Module^(37)^ and demographic module in the participant survey were discussed, and tailoring to specific subpopulations was proposed as an overarching solution. Language translation, alongside any tailoring, was key to increasing comprehension. In the past, the NTAE has published specific survey development and administration strategies to address raised issues^(41)^. These include development of measures that include interchangeable food examples, new fruit and vegetable categories or types, valid language translation instructions for spoken and written word, within language translation that incorporates cross-cultural differences; test for and avoiding stigmatising language, collecting qualitative and quantitative data, validating measures across and within subpopulations, collapsing response options and offering ‘don’t know’ and ‘prefer not to answer’ response options. Given the tension between the need to validate survey tools and the urgency to tailor them to better reflect intervention and health outcomes, specific funding mechanisms should be dedicated to validating measures and analytical approaches to reflect various populations.

Acknowledging similar or different identities between the survey participant and data collector was highlighted as valuable for the data collection process. Age, gender, race, ethnicity, profession, language and appearance influenced how the survey participant and data collector interacted within a community. Partnering with the community to plan data collection, hiring local data collectors, partnering with community health workers, providing appropriate compensation, training data collectors, collecting data in person and being intentional about times and locations of data collection were mentioned as key to enhancing the participant’s experience in research. Additionally, being purposeful about building rapport with the participant through icebreaker conversation or questions when recruiting for survey participants was reported to help enhance trust during the data collection process^(42)^.

The challenges and opportunities highlighted in this study offer valuable insights that align with broader issues found in public health survey research, including nutrition-related studies. For example, calls for increasing the linguistic diversity of the USDA ERS Household Food Security Survey Module have been made over time to ensure understanding across communities^(43–45)^. In another example, the US Census has faced recent challenges in classifying racial and ethnic categories with regard to the Middle Eastern or North African^(46)^. This study’s findings illuminate that adaptation and ongoing validation of public health survey tools and methods are necessary to effectively represent populations. Furthermore, the approaches used to identify challenges and opportunities for data collection are valuable for improving these processes. Limitations in this study include that while interview and focus group participants worked across populations, not every ethnic and racial group was represented fully. For instance, although the participant-level GusNIP survey sample size was large, Asian, Native Hawaiian and other Pacific Islander populations were a smaller part of the overall GusNIP sample as compared White and Black or African American participants. Thus, challenges and opportunities are not exhaustive. Research participants were not reflective of survey participants in GusNIP projects – for example, research participants were under the age of 55. Future research should focus on the needs and strategies that emerge from specific subpopulations to enhance generalisability. As in all qualitative research, interview and focus group participants contributed to various degrees. Other complementary research could collect quantitative survey data from a larger set of participants.

### Conclusion

It is critical to accurately represent the outcomes and experiences across populations in public health research. To do so, evaluation must include tools and approaches that are understood and accepted across and within subpopulations. Engaging affected populations and communities of public health nutrition projects is key to ensure accurate representation. The challenges and opportunities identified about the GusNIP NTAE’s participant survey can be applied to other public health nutrition interventions – this research provides foundational information to improve evaluation toward better understanding outcomes across participants and inform implementation strategies of interventions.

## References

[1] U.S. Department of Agriculture & U.S. Department of Health and Human Services (2020) Dietary Guidelines for Americans, 2020–2025. https://www.dietaryguidelines.gov. Accessed February 2025.

[2] Jemal A , Ward E , Anderson RN et al. (2008) Widening of socioeconomic inequalities in U.S. death rates, 1993–2001. PLOS ONE 3, e2181. 10.1371/journal.pone.0002181 18478119 PMC2367434

[3] Bell CN , Thorpe RJ , Bowie JV et al. (2018) Race disparities in cardiovascular disease risk factors within socioeconomic status strata. Ann Epidemiol 28, 147–152. 10.1016/j.annepidem.2017.12.007 29317176

[4] Mariotto AB , Zou Z , Johnson CJ et al. (2018) Geographical, racial and socio-economic variation in life expectancy in the US and their impact on cancer relative survival. PLOS ONE 13, e0201034. 10.1371/journal.pone.0201034 30044829 PMC6059474

[5] Skolarus LE , Sharrief A , Gardener H et al. (2020) Considerations in addressing social determinants of health to reduce racial/ethnic disparities in stroke outcomes in the United States. Stroke 51, 3433–3439. 10.1161/STROKEAHA.120.030426 33104471 PMC7732185

[6] Centers for Disease Control and Prevention (2024) National Diabetes Statistics Report. 2024, January 8. https://www.cdc.gov/diabetes/php/data-research/index.html. Accessed February 2025.

[7] Bowleg L (2012) The problem with the phrase women and minorities: intersectionality—an important theoretical framework for public health. Am J Public Health 102, 1267–1273. 10.2105/AJPH.2012.300750 22594719 PMC3477987

[8] Bauer GR (2014) Incorporating intersectionality theory into population health research methodology: challenges and the potential to advance health equity. Social Sci Med 110, 10–17. 10.1016/j.socscimed.2014.03.022 24704889

[9] Satia JA (2009) Diet-related disparities: understanding the problem and accelerating solutions. J Am Dietetic Assoc 109, 610–615. 10.1016/j.jada.2008.12.019 PMC2729116 19328255

[10] Healthy People 2030 (n.d) Social Determinants of Health. https://health.gov/healthypeople/priority-areas/social-determinants-health. Accessed February 2025.

[11] Zhang FF , Liu J , Rehm CD et al. (2018) Trends and disparities in diet quality among US adults by Supplemental Nutrition Assistance Program participation status. JAMA Network Open 1, e180237. 10.1001/jamanetworkopen.2018.0237 30498812 PMC6258006

[12] Hiza HAB , Casavale KO , Guenther PM et al. (2013) Diet quality of Americans differs by age, sex, race/ethnicity, income, and education level. J Acad Nutr Diet 113, 297–306. 10.1016/j.jand.2012.08.011 23168270

[13] Lee SH , Moore LV , Park S et al. (2022) Adults meeting fruit and vegetable intake recommendations—United States, 2019. Morbidity Mortality Weekly Rep 71, 1–9. 10.15585/mmwr.mm7101a1 PMC8735562 34990439

[14] Kaiser Family Foundation (2022) Poverty Rate by Race/Ethnicity. KFF. https://www.kff.org/other/state-indicator/poverty-rate-by-raceethnicity/. Accessed March 2025.

[15] Shrider EA , Kollar M , Chen F et al. (2021) Income and Poverty in the United States: 2020. United States Census Bureau; 2021(92). 2021, September. https://www.census.gov/content/dam/Census/library/publications/2021/demo/p60-273.pdf. Accessed March 2025.

[16] Rabbitt MP , Hales LJ , Burke MP et al. (2023) Household Food Security in the United States in 2022. U.S. Department of Agriculture Economic Research Service. 2023, October. http://www.ers.usda.gov/publications/pub-details/?pubid=107702. Accessed March 2025.

[17] U.S. Department of Agriculture (2024) What is Nutrition Security? Food and Nutrition Security. https://www.usda.gov/nutrition-security. Accessed February 2025.

[18] U.S. Department of Agriculture National Institute of Food and Agriculture (2024) Gus Schumacher Nutrition Incentive Program—Nutrition Incentive Program (GusNIP-NI). https://www.nifa.usda.gov/gusnip-request-applications-resources-ni. Accessed February 2025.

[19] U.S. Department of Agriculture National Institute of Food and Agriculture (2024) Gus Schumacher Nutrition Incentive Program (GusNIP). https://www.nifa.usda.gov/grants/programs/hunger-food-security-programs/gus-schumacher-nutrition-incentive-program. Accessed February 2025.

[20] U.S. Department of Agriculture National Institute of Food and Agriculture (2024) Gus Schumacher Nutrition Incentive Program—Produce Prescription (GusNIP-PPR). https://www.nifa.usda.gov/gusnip-request-applications-resources-ppr. Accessed February 2025.

[21] U.S. Department of Agriculture National Institute of Food and Agriculture (2024) Gus Schumacher Nutrition Incentive Program—National Training, Technical Assistance, Evaluation, and Information Centers Program (GusNIP-NTAE). https://www.nifa.usda.gov/gus-schumacher-nutrition-incentive-program-national-training-technical-assistance-evaluation. Accessed February 2025.

[22] Barth MM , Bell RA & Grimmer K (eds) (2020) Public Health Nutrition: Rural, Urban, and Global Community-Based Practice, Chapter 13. New York: Springer Publishing Company.

[23] Ward M , Schulz AJ , Israel BA et al. (2018) A conceptual framework for evaluating health equity promotion within community-based participatory research partnerships. Evaluation Program Planning 70, 25–34. 10.1016/j.evalprogplan.2018.04.014 29894902 PMC6077092

[24] Stern S , Guckenburg S , Persson H et al. (2019) Reflections on Applying Principles of Equitable Evaluation. Justice, Prevention Research Center. https://www.wested.org/wp-content/uploads/2019/07/resource-reflections-on-applying-principles-of-equitable-evaluation.pdf. Accessed February 2025.

[25] Equitable Evaluation Initiative (2023) EEF Expansion: Elements of the EEF-Principles. https://www.equitableeval.org/post/eef-expansion-principles. Accessed February 2025.

[26] Gutuskey L (2022) Centering Equity in Program Evaluation. Administration for Children & Families, U.S. Department of Health and Human Services. https://www.acf.hhs.gov/sites/default/files/documents/opre/centering_equity_program_evaluation_feb2023.pdf. Accessed February 2025.

[27] Office of the Assistant Secretary for Planning and Evaluation & U.S. Department of Health and Human Services (2022) Equitable Evaluation Series: Principles of Equitable Evaluation. https://aspe.hhs.gov/sites/default/files/documents/d4bfa84a6fc3ac13904525c86e1078ee/ees-principles-of-equitable-communication.pdf. Accessed February 2025.

[28] Dhillon J , Jacobs AG , Ortiz S et al. (2022) A Systematic review of literature on the representation of racial and ethnic minority groups in clinical nutrition interventions. Adv Nutr 13, 1505–1528. 10.1093/advances/nmac002 35108358 PMC9526835

[29] Russell M (2019) To support all: diversity and inclusion. J Acad Nutr Diet 119, 543. 10.1016/j.jand.2019.02.001 30905427

[30] North Carolina Center for Nonprofits (2024) Open Source Leadership Strategies. https://www.ncnonprofits.org/. Accessed February 2025.

[31] Equitable Evaluation Initiative (2024) The Equitable Evaluation Framework (EFF) is a Set of Principles, Orthodoxies, Mindsets, Tensions, and Sticking Points. Framework. https://www.equitableeval.org/framework. Accessed February 2025.

[32] Mensch L & Souza K (2021) An Introduction to Incorporating Diversity, Equity, and Inclusion into Nutrition Incentive Program Research and Evaluation. Nutrition Incentive Hub. 2021, February. https://www.nutritionincentivehub.org/media/rliah2qb/mensch_souza_msu-crfs_dei-in-ni-research-and-evaluation_2021-02.pdf. Accessed February 2025.

[33] Story M , Kaphingst KM , Robinson-O’Brien R et al. (2008) Creating healthy food and eating environments: policy and environmental approaches. Ann Rev Public Health 29, 253–272. 10.1146/annurev.publhealth.29.020907.090926 18031223

[34] Gus Schumacher Nutrition Incentive Program National Training, Technical Assistance, Evaluation, and Information Centers Program (GusNIP NTAE) (2023) GusNIP NTAE Y3 Impact Findings: September 1, 2012 to August 31, 2022. https://nutritionincentivehub.org/gusnip-ntae-y3-impact-findings. Accessed February 2025.

[35] Budd Nugent N , Byker Shanks C , Seligman HK et al. (2021) Accelerating evaluation of financial incentives for fruits and vegetables: a case for shared measures. Int J Environ Res Public Health 18, 12140. 10.3390/ijerph182212140 34831902 PMC8621044

[36] Charmaz K (2006) Constructing Grounded Theory: A Practical Guide through Qualitative Analysis. London: Sage Publications.

[37] Bickel G , Nord M , Price C et al. (2000) Guide to Measuring Household Food Security. U.S. Department of Agriculture Food and Nutrition Service. https://nhis.ipums.org/nhis/resources/FSGuide.pdf. Accessed February 2025.

[38] Guest G , MacQueen KM & Namey EE (2011) *Applied Thematic Analysis*. Sage Publications. 10.4135/9781483384436

[39] National Institutes of Health National Cancer Institute Division of Cancer Control & Population Studies (2024) Dietary Screener Questionnaire (DSQ) in the NHANES 2009–10: Dietary Factors, Food Items Asked, and Testing Status for DSQ. https://epi.grants.cancer.gov/nhanes/dietscreen/evaluation.html#pub. Accessed February 2025.

[40] Baillie E , Bjarnholt C , Gruber M et al. (2009) A capacity-building conceptual framework for public health nutrition practice. Public Health Nutr 12, 1031–1038. 10.1017/S1368980008003078 18940028

[41] Byker Shanks C , Parks CA , Izumi B et al. (2022) The need to incorporate diversity, equity, and inclusion: reflections from a national initiative measuring fruit and vegetable intake. J Academy Nutr Diet 122, 1241–1245. 10.1016/j.jand.2022.01.011 35077871

[42] Bell K , Fahmy E & Gordon D (2016) Quantitative conversations: the importance of developing rapport in standardised interviewing. Qual Quantity 50, 193–212. 10.1007/s11135-014-0144-2 PMC4705135 26792949

[43] Kwan CM , Napoles AM , Chou J et al. (2015) Development of a conceptually equivalent Chinese-language translation of the US Household Food Security Survey Module for Chinese immigrants to the USA. Public Health Nutr 18, 242–250. 24642365 10.1017/S1368980014000160 PMC4169349

[44] Rabbitt MP & Coleman-Jensen A (2017) Rasch analyses of the standardized Spanish translation of the US Household Food Security Survey Module. J Econ Social Meas 42, 171–187.

[45] Stokes-Ramos H (2024) Beyond economic barriers: conceptualizing food insecurity among resettled refugees living in the United States. J Immigrant Refugee Stud 1–19.

[46] Marks R , Jones N & Battle K (2024) What Updates to OMB’s Race/Ethnicity Standards Mean for the Census Bureau. Random Samplings. Suitland, Maryland: United States Census Bureau; available at https://www.census.gov/newsroom/blogs/random-samplings/2024/04/updates-raceethnicity-standards.html. Accessed February 2025.

